# Perspectives of continuous renal replacement therapy in the intensive care unit: a paired survey study of patient, physician, and nurse views

**DOI:** 10.1186/s12882-015-0086-5

**Published:** 2015-07-14

**Authors:** Andrew S. Allegretti, Gregory Hundemer, Rajeev Chorghade, Katherine Cosgrove, Ednan Bajwa, Ishir Bhan

**Affiliations:** Divsion of Nephrology, Department of Medicine, Massachusetts General Hospital, 7 Whittier Place, Suite 106, Boston, 02114 MA USA; Department of Medicine, Massachusetts General Hospital, Boston, MA USA; Divsion of Pulmonary and Critical Care Medicine, Department of Medicine, Massachusetts General Hospital, Boston, MA USA

**Keywords:** Continuous renal replacement therapy, Intensive care unit, Kidney injury, Mismatch, Patient and provider perspectives, Survey

## Abstract

**Background:**

Recent studies suggest discrepancies between patients and providers around perceptions of hemodialysis prognosis. Such data are lacking for continuous renal replacement therapy (CRRT). We aim to assess patient and provider understanding of outcomes around CRRT.

**Methods:**

From February 1 to August 31, 2013, a triad of (1) a patient on CRRT (or health care proxy [HCP]), (2) physician and (3) primary nurse from the intensive care unit (ICU) team were surveyed. Univariate chi-square and qualitative analysis techniques were used.

**Results:**

Ninety-six total participants (32 survey triads) were completed. Ninety one percent of patients/HCPs correctly identified that CRRT replaced the function of the kidneys. Six percent of patients/HCPs, 44 % of physicians, and 44 % of nurses identified rates of survival to hospital discharge that were consistent with published literature. Both physicians and nurses were more likely than patients/HCPs to assess survival consistently with published data (p = 0.001). Patients/HCPs were more likely to overestimate survival rates than physicians and nurses (p < 0.001). Thirty eight percent of patients/HCPs, 38 % of physicians, and 28 % of nurses identified rates of lifelong dialysis-dependence among surviving patients that were consistent with published literature.

**Conclusions:**

There is mismatch between patients, HCPs, and providers around prognosis of CRRT. Patients/HCPs are more likely to overestimate chances of survival than physicians or nurses. Further intervention is needed to improve this knowledge gap.

**Electronic supplementary material:**

The online version of this article (doi:10.1186/s12882-015-0086-5) contains supplementary material, which is available to authorized users.

## Background

Continuous renal replacement therapy (CRRT) is often employed in hemodynamically unstable patients in the intensive care unit (ICU) with severe acute kidney injury (AKI) and end stage renal disease (ESRD). [1, 2] In-hospital mortality in this patient population is high, consistently reported as over 50 %. [3–7] However, patients with severe AKI that survive their hospital stay have an excellent chance of recovering renal function; over 85 % of these patients do not require indefinite renal replacement therapy [3, 7, 8].

An accurate understanding of prognosis allows patients and their health care proxies (HCPs) to make informed decisions about goals of care. This understanding is critical to maintain good patient-provider relationships, and may be associated with less psychosocial distress for patients and HCPs. [9–11] The majority of studies assessing patient and provider expectations of illness are in oncologic disease. These data suggests that both groups overestimate likelihood of survival, with patients being more optimistic than their physicians overall. [12–16] Similarly, a recent survey study among nephrologists and patients with ESRD showed that patients dramatically overestimate their probability of long-term survival and were more optimistic about their candidacy for renal transplantation compared to their physicians [17].

Maintaining effective communication and making shared treatment decisions pose many challenges in the ICU setting. [18–23] The initiation of CRRT presents an opportunity for patients/HCPs and ICU providers to discuss the logistics and implications of starting a new procedure and often leads to questions about expectations of illness. Despite many attempts to identify potential risk factors for poor outcomes in this patient population, there is no widely accepted predictive model, and thus the potential for communication mismatch exists. [3, 24, 25] There are currently no studies in the literature that assess the expectations of survival and renal recovery of patients/HCPs, physicians, and critical care nurses around patients receiving CRRT. We sought to assess this potential mismatch of the prognostic assessment between these groups using a survey tool that assesses all three groups.

## Methods

### Setting and Participants

Patients who were treated with CRRT in the medical or cardiac ICUs at Massachusetts General Hospital (MGH) between February and August 2013 were eligible for this study. Patients were excluded if the primary medical team did not feel that a survey study was appropriate given the clinical context of the patient’s care (*e.g.*, if patient’s care was limited to comfort measures). Surveys were performed within the first 48 hrs of a patient starting CRRT. Surveys were administered either verbally or on paper depending on participant preference. If patients were unable to complete the survey (due to their medical situation, *e.g.* intubation or sedation), their HCPs were interviewed instead. No member of the research team was directly involved in patient care for any of the participants in this study. All surveys were performed in English and all respondents self-reported fluency in English.

MGH is a tertiary care hospital with 1008 total beds and 126 ICU beds available for CRRT, 34 of which are in the medical or cardiac ICU. CRRT is provided as continuous veno-venous hemofiltration using machines from NxStage Medical (Lawrence, MA). All nurses who perform CRRT have received specialized training and have at least 18 months of critical care nursing experience at MGH. Decisions to initiate CRRT are made by nephrologists in consultation with intensivists. CRRT is performed in lieu of intermittent hemodialysis as per local standard of care. Consent and education about CRRT was performed as per standard of care by the consulting nephrology team prior to the initiation of CRRT. No formal script or additional educational material around CRRT was provided to those participating in this study by the study team.

### Survey Methodology

A member of the research team identified potential index patient cases who were initiated on CRRT within 48 hrs by reviewing the ICU and nephrology consult censuses. Each survey triad included (1) a patient on CRRT (or their HCP), (2) an ICU physician from the primary care team, and (3) an ICU nurse currently employing CRRT for that patient. Patients/HCPs were given a six question survey designed by the research team that included one open ended question, two “true or false” questions, and three multiple choice questions (Table 1). Surveys assessed understanding of CRRT (questions 1–3), expectations of survival and need for indefinite dialysis (questions 4–5), and how well the care team explained CRRT (question 6). In order to maximize comparability within survey triads, multiple-choice questions were presented as four choices of percentage quartiles. Following each patient/HCP survey, a member of the primary medical ICU team and a primary nurse were independently asked three analogous questions about general expectations of survival, likelihood of requiring indefinite dialysis, and assessment of patient/HCP understanding of CRRT. All participants were asked to give generalized answers about patients on CRRT rather than the index case in order to minimize bias. Providers who cared for more than one patient enrolled in this study were only surveyed once. Sufficient survey-naïve providers were available for all index cases. Physicians surveyed were critical care attendings, critical care or cardiology fellows, or internal medicine trainees (post graduate year one through four) and were selected based on survey-naïve status and clinical availability. Medical records were reviewed for relevant demographic and medical information about the patient’s admission. Primary admitting diagnosis for each patient was determined from the medical record discharge summary.

**Table 1:** Survey – Subjects were asked one open-ended question (question 1, patient/HCP only) and five multiple-choice questions (questions 1–6)

Number	Question	Response
1	What is your understanding of this machine’s purpose?	Open Ended
		Patient/HCP only
2	CRRT is a procedure done on patients who have kidney failure where a machine replaces the job of the kidneys.	True or False
		Correct Answer: True
		Patient/HCP only
3	CRRT helps the kidneys heal faster	True or False
		Correct Answer: False
		Patient/HCP only
4	How many patients who are treated with CRRT in the intensive care unit will need to be on dialysis forever after leaving the hospital?	Multiple Choice
		Choices: <25 %, 25-49 %, 50-75 %, ≥ 75 %
5	How many patients who are treated with CRRT in the intensive care unit will survive and leave the hospital?	Multiple Choice
		Choices: <25 %, 25-49 %, 50-75 %, ≥ 75 %
6	*For Patients/HCP*: How well did the doctors do in explaining why I (or my family member) needs CRRT?	Multiple Choice
	*For MD and Nurse*: How well do you feel the patient or HCP understands why they need CRRT?	Choices: Completely, mostly, a little, not at all

### Statistical Analysis

A prior study at MGH assessing all patients requiring CRRT from 2008 to 2011 reported the probability of survival to hospital discharge between 25 % and 50 % and the probability of requiring indefinite dialysis once discharged at less than 25 %. [3] These results are supported by multiple prior studies. [4, 5, 7, 8, 26–34] These responses were considered consistent with the literature when adjudicating responses.

Immersion crystallization and codebook analysis were performed to identify important and recurring themes for open-ended answers in question 1. [35, 36] Descriptive analysis was performed for questions 2, 3, and 6. Univariate Fisher’s exact analysis was performed for questions 4 and 5 to compare responses between patient/HCP, physician, and nurse subgroups. Univariate rank sum and Fisher’s exact analyses were used for continuous and categorical variables, respectively, to analyze for correlation between patient/provider demographic or medical background information and correct answer choices. STATA version 12.1 (StataCorp LP, College Station, TX) was used for all statistical analysis.

A pre-determined interim analysis was performed after 20 survey triads were completed. After an additional 12 triads were completed without significant change to preliminary results study recruitment was deemed complete.

### Ethics Statement

Written informed consent was obtained from all study participants. All responses and patient/provider information were de-identified except to members of the research team. The Partners Human Research Committee for human subjects approved the study. All clinical investigation was conducted according to the principles expressed in the Declaration of Helsinki.

## Results

### General Demographics

Ninety-six participants (32 survey triads) were interviewed for this study. Twenty-three patients (72 %) were hospitalized in a medical ICU and nine (28 %) were hospitalized in a cardiac ICU. Twenty-five patients (78 %) required CRRT for AKI and the remaining 7 patients (22 %) had a diagnosis of ESRD. The most common primary diagnoses for ICU admission were sepsis/infection (38 %), cardiogenic shock (16 %), and liver disease/cirrhosis (16 %). From the patient/HCP subgroup, the vast majority of responders were HCPs (88 %). Of the four patients who responded themselves, three had a diagnosis of ESRD. Eighteen of the 32 patients (56 %) survived to hospital discharge. Of patients with AKI who survived to discharge, twelve of the 15 patients (80 %) recovered renal function (as defined by not requiring any renal replacement therapy at discharge). Physicians surveyed were attending physicians (22 %), critical care or cardiology fellows (13 %), or medical residents (66 %) between post graduate years one and four of residency training (Table 2).

**Table 2:** Demographics

Patient/HCP Demographics	
Age, mean (range, standard deviation)	56 years (35–90 years, 14)
Patient (n = 4/32)	61 years (35–90 years, 14)
HCP (n = 28/32)	55 years (33–80 years, 14)
Male Gender	21 (66 %)
Reason for CRRT	
AKI	25 (78 %)
ESRD	7 (22 %)
Race	
White	23 (72 %)
Black	4 (13 %)
Non-black Hispanic	3 (9 %)
Other	2 (6 %)
Highest Level of Education	
Did not complete high school	1 (3 %)
Completed high school	11 (34 %)
Partial college	6 (19 %)
Completed college	10 (31 %)
Post-graduate	4 (13 %)
Patient Location	
Medical ICU	23 (72 %)
Cardiac ICU	9 (28 %)
Primary Diagnosis	
Sepsis/Infection	12 (38 %)
Cardiogenic Shock	5 (16 %)
Liver disease/Cirrhosis	5 (16 %)
Pancreatitis	2 (6 %)
Hemorrhagic Shock	2 (6 %)
Other	6 (19 %)
Survived to Discharge (Patients)	18 (56 %)
Recovered Renal Function (of n = 15 AKI patients who survived to discharge)	12 (80 %)
Level of Training of MDs	
Attending	7 (22 %)
Fellow	4 (13 %)
Resident	21 (66 %)
Post graduate year (PGY) 1	3 (9 %)
PGY 2	9 (28 %)
PGY 3 or 4	9 (28 %)

All data for patient/HCP categories correspond to the survey responder unless otherwise noted

### Closed-ended Survey Results

When asked true/false questions describing the procedure, 91 % (n = 29/32) of patients/HCPs correctly identified the purpose of CRRT. Fifty three percent (n = 17/32) of patients/HCPs incorrectly thought CRRT improved the speed of renal recovery.

**Fig. 1:**
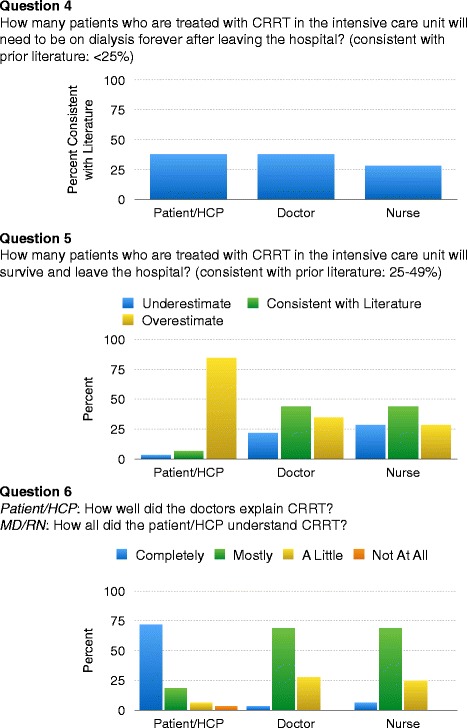
Perceptions of CRRT outcomes. Patients and/or health care proxies (HCP) were more likely to overestimate survival following continuous renal replacement therapy (CRRT) and had a more positive view on provider/patient communication than physicians or nurses

In multiple-choice format, 6 % of patients/HCPs (n = 2/32), 44 % of physicians (n = 14/32), and 44 % of nurses (n = 14/32) identified rates of survival to hospital discharge consistently with published literature (25-49 %). Both physicians (p = 0.001) and nurses (p = 0.001) were more likely than patients/HCPs to assess survival consistently with published data. Eighty-four percent (n = 27/32) of patients/HCPs overestimated their likelihood of survival by answering “≥75 %” (n = 18/32) or “50-74 %” (n = 9/32). Thirty-four percent (n = 11/32) of physicians and 25 % (n = 8/32) of nurses overestimated probability of survival compared to the literature. Patients/HCPs were significantly more likely to overestimate probability of survival compared to physicians (p < 0.001) and nurses (p = <0.001; Fig. 1). In a secondary analysis including a wider range of survival rates as consistent with the literature (25-49 % and 50-74 %), physicians and nurses remained more likely to assess survival consistently with published data compared to patients/HCPs (p = 0.005 and p = 0.01, respectively). Within the patient/HCP subgroup, responses from patient respondents, children-HCPs and spouse-HCPs were similar, as were responses for those with AKI compared to ESRD.

In multiple-choice format, 38 % of patients/HCPs (n = 12/32), 38 % of physicians (n = 12/32), and 28 % of nurses (n = 9/32) identified the probability of requiring lifelong dialysis if discharged consistently with published literature (less than 25 %). There were no significant differences between these three subgroups for this question (p = 0.69; Fig. 1). No demographic factors correlated with responses for renal recovery or survival in any of the three subgroups. Within the physician subgroup, responses from residents, fellows, and attending physicians were similar. Within the patient/HCP subgroup, responses from patient respondents, children-HCPs and spouse-HCPs were similar, as were responses for those with AKI compared to ESRD.

The majority of patients/HCPs answered that their doctors “completely” explained the purpose of CRRT (72 %, n = 23/32). In comparison, physicians and nurses felt that patients/HCPs “mostly” understood the role of this procedure (69 %, n = 22/32 for both subgroups), while only one physician (3 %) and two nurses (6 %) agreed that the patient/HCP had “complete” understanding (Fig. 1).

### Open-ended Survey Results

In question 1, patients/HCPs were asked to describe the purpose of CRRT with an open-ended response (Additional file 1: Table S1). Responses were varied and covered several thematic areas. The most common motif expressed was CRRT “cleaning” or “filtering” the blood from toxins, which was present in 15 responses:

“…I know the blood is coming out, being cleansed, and going back in - that’s why there are two lines.”

Five patients/HCPs identified CRRT as a replacement for the native kidney. Three patients made comparisons to intermittent HD (all three were initiated on CRRT for acute kidney injury rather than for ESRD). Ten patients/HCPs commented on the speed or duration of CRRT, describing it as continuous, slower, or gentler than alternative forms of hemodialysis:

“My understanding was that it was used to clean the toxins from the blood system. This is a slower machine than all the hospitals have. His blood pressure couldn’t take the stress of the other machine.”

Four patients recognized CRRT was an extracorporeal therapy and that blood was removed and then returned to the body, with three additional patients acknowledging that this process would also remove fluid. Two patients noted that they did not feel like they understood the machine (both correctly answered the corresponding true/false question about the machine’s purpose). Several patients exhibited some level of misunderstanding about the CRRT, most commonly stating that the machine “fixed” or “repaired” the kidneys, or giving the machine credit for performing a function that it does not do:

“To use the machine to help kick the kidneys to produce urine…”

“The setup allows dialysis to occur in slow but direct methods in which blood is pumped directly into her to deliver medicine more quickly and directly.”

## Discussion

Our study describes the single-center experience of mismatch around patient/HCP and provider perceptions of CRRT using a non-validated survey tool and descriptive analysis techniques. Of note, most patients in this cohort were too ill to participate in a survey, and therefore 88 % of responders from the patient/HCP group were HCPs. While HCPs often must make decisions for patients in the ICU, it should be noted that HCPs represented the majority of the patient/HCP subgroup, and may not necessarily have the same views or understanding as the patients themselves.

One of the most prominent findings in this study was that all three subgroups – patients/HCPs, ICU physicians, and nurses – provided answers that were inconsistent with published literature around survival and likelihood of requiring long-term dialysis. Forty-four percent of ICU physicians and nurses identified in-hospital mortality rates consistently with the literature, as did just 6 % of patients/HCPs. All three subgroups were similarly discrepant in assessing the likelihood of requiring long-term dialysis compared to prior studies. While both nurses and physicians were significantly more consistent with published probabilities of survival than patients/HCPs, these responses highlight a need for further education for patients and providers alike. Our results are consistent with prior literature examining attending and resident physicians’ accuracy of prognosis. Survey studies around patients with acute congestive heart failure, late stage cancer, and critical illness requiring ICU admission described similarly low rates of prognostic accuracy. [13, 37–39] At our study site, consulting nephrologists, rather than ICU physicians or nurses, obtain informed consent for CRRT. This study did not assess the degree of communication between consulting nephrologists and the ICU care team and patients/HCPs. The consent process is a natural time for patients/HCPs to ask questions about survival and potential long-term dialysis needs; this interaction may serve as another potential target for investigation of perceptions of CRRT.

This study included only patients who were initiated on CRRT, as opposed to intermittent hemodialysis. While studies comparing outcomes between these two populations have shown similar rates of survival and long-term dialysis dependence, [40] the results of this study are best generalized to the CRRT population. It should also be noted that the 97 % of patient/HCPs identified as having a high school education or greater. Those with lower education levels might have even worse prognostic estimates, though this conjecture requires further study to evaluate in the CRRT population.

Compared to their physicians, our results show that patients/HCPs overestimate the chance of survival, which is consistent with prior literature comparing patient and provider views in the non-ICU setting. [12–17] Our study is novel in that it also examines ICU nurses’ perceptions of patient outcomes. Despite the fact that nurses have more direct contact with patients/HCPs than physicians, [41, 42] we found no differences in the responses of physicians and nurses. Nurses’ understanding of illness may have a strong impact on patient/HCP perceptions and expectations, and thus highlights an additional opportunity for interventions to improve understanding of prognosis and communication in the ICU. This concept requires further directed study to examine the impact of nursing beliefs of illness on care of patients with CRRT.

Perhaps the richest patient/HCP information came in open-ended responses to question 1. Overall, responses spanned several thematic areas important to the function of CRRT. With few exceptions, patients/HCPs were generally accurate in describing some of the reasons CRRT was employed. Answers were typically short, and very few patients talked about CRRT as a life-supporting measure. Answers typically focused on the mechanical function of the device – commonly described as a filter, a “slow” treatment, or as an extracorporeal device with blood being removed then returned to the body. It is not known if this pattern of responses was because the idea of CRRT as life support (analogous to a ventilator, for example) was not emphasized during the consent or patient education process, or because this was difficult of patient/HCPs to acknowledge.

There is an interesting paradox that exists in the literature surrounding communication of advanced illness. Even after candid conversations about advanced care planning, patient/HCP understanding of prognosis and surrogate decision-making preferences has been shown to be low. [43, 44] However, recent data suggests that many patients prefer to hear from their doctor if their overall prognosis is poor, suggesting that providers should feel both empowered and obligated to share as much prognostic information as appropriate with patients and HCPs. [45, 46] These discussions can be challenging in the ICU setting, however, due to lack of consistent prediction models, frequently evolving treatment courses, variations in patient/HCP belief systems, and the emotional stresses inherent to the ICU. [3, 18–25] At this study site, there is no standardized patient education process around CRRT, which may be a barrier to improving discordance around CRRT. Augmenting direct clinician counseling with multimedia educational tools, such as decision support videos, has been shown to be successful in improving patient/HCP understanding of illness and assisting in making decisions consistent with patients’ value systems. [47] These tools have been used in other patient populations with poor overall prognosis, including counseling about cardiopulmonary resuscitation in the ICU. [48–53] Our study highlights a significant knowledge gap and communication mismatch between patients/HCPs and ICU providers around CRRT, suggesting that there may be a role for such a tool around this intervention.

This study should be interpreted with the context of its limitations. It was performed at a single site using a small sample, thus potentially limiting generalizability. Since this study was not powered to pick up individual predictors of accuracy, no patient or HCP demographic factors correlated with answer choices. However, this study was designed in large part to be a descriptive and qualitative investigation of perceptions of illness across parallel patient and provider groups. We feel that our holistic message is more important than the power of the individual data points. ICU physicians and nurses were instructed to answer based on perceptions of overall survival and renal recovery rates (rather than for the index patient case) in order to maximize objectivity and comparability between groups. However, provider expectations for each index patient may have had an influence on answer choices due to temporal proximity to the survey, thus capturing some degree of bias. In line with this, the survival rate in this sample was 59 %, which is higher than the rates of less than 50 % that are routinely published in the literature. This is likely explained by a selection bias, as sicker patients who had a higher mortality were more likely to die (or transitioned to comfort care measures) prior to being interviewed, thus artificially improving survival in our sample. Still, this may have affected responses in all three subgroups. Because no validated survey tool previously existed that could assess the questions of this study, our research group had to create our own short survey. Prior studies assessing patient understanding of illness in hemodialysis and in the ICU have done so with similar, non-validated survey tools, [17, 50] so we feel that are results remain interpretable. Prior to the beginning of the study, the survey tool was reviewed by two members of each subgroup in order to ensure understandability. These subjects were not included in the final analysis.

## Conclusions

There is mismatch between patients/HCPs and ICU providers around prognosis of CRRT. Compared to physicians and nurses, patients/HCPs are more likely to overestimate the likelihood of survival on CRRT. Patients/HCPs may overestimate their level of understanding of CRRT, and may believe that CRRT improves the speed of renal recovery. New strategies are needed in order to minimize this discordance and improve the use of CRRT within the framework of patient-centered treatment plans.

## Additional file

Additional file 1:
**Patient/HCPs Responses to Question One: What is your understanding of this machine’s purpose?**

## Abbreviations

AKI: Acute Kidney Injury
CRRT: Continuous Renal Replacement Therapy
ESRD: End Stage Renal Disease
HCP: Health Care Proxy
ICU: Intensive Care Unit
MGH: Massachusetts General Hospital
