# Supplementary material for: Perspectives of continuous renal replacement therapy in the intensive care unit: a paired survey study of patient, physician, and nurse views
Source: BMC Nephrol. 2015 Jul 14;16:105. doi: 10.1186/s12882-015-0086-5 (PMC4501124; doi:10.1186/s12882-015-0086-5)
Supplement: Additional file 1: — Patient/HCPs Responses to Question One: What is your understanding of this machine’s purpose? [file 12882_2015_86_MOESM1_ESM.docx]

**Supplementary Table 1: Patient/HCPs Responses to Question One: What is your understanding of this machine’s purpose?**

| **Patient/HCP** | **Response** |
| --- | --- |
| 1 | This will continuously remove the blood and filter and clean it the way the kidney would do, then put the blood back in. |
| 2 | To clean his blood and to draw fluid from his lungs. More specifically, to draw fluid out of his body so it can get out of his lungs. It is a slower rate than his usual dialysis |
| 3 | I know about fistulas…I know the blood is coming out, being cleansed, and going back in - that's why there are two lines. It's continuous, which is different from the three times a week kind. You know, some people do it at home now, which is really cool. |
| 4 | Her kidneys are not functioning and this machine will alleviate that problem |
| 5 | This machine is to assist in his kidneys by providing continuous but gentle cleaning. |
| 6 | Help her system recover from shock after the incident and the clear anesthetic and paralytics after she was cooled |
| 7 | Not much. It's keeping me alive. It's working for my kidneys. |
| 8 | To take over the job of the kidney. |
| 9 | I believe the machine filters the blood out, removes the impurities, then returns the blood. |
| 10 | To get the kidney working. |
| 11 | To clean the blood of toxins. |
| 12 | I don't know, dialysis I guess. The doctors never really explained it to me. |
| 13 | Cleanse the blood in place or in conjunction with the kidneys |
| 14 | Dialysis |
| 15 | Filters the blood of toxins |
| 16 | To provide 24 hour dialysis |
| 17 | Filtration of blood, basically taking over kidney functions. |
| 18 | To clean up the blood and clean the kidneys |
| 19 | It's a new machine that they are trying out because the dialysis machine they were using previously wasn't doing a good job. This one is doing a good job. |
| 20 | Her kidneys shut down and they are cleaning the poisons in her body. It's like a dialysis machine. They will take her blood out and put clean blood in. |
| 21 | For slow dialysis instead of the quick dialysis |
| 22 | To use the machine to help kick the kidneys to produce urine and a life support system. |
| 23 | Helping kidneys work while his kidneys heal and filtering the blood |
| 24 | Supports his kidneys |
| 25 | The continuous dialysis machine cleans blood at a slow pace. The rate of blood flow must be slow so that it can clean continuously. I believe it cleans more thoroughly than the 3-5 hour treatments. |
| 26 | To help his kidneys work better and help his breathing, to help blood flow through his kidneys for oxygen. |
| 27 | The setup allows dialysis to occur in slow but direct methods in which blood is pumped directly into her to deliver medicine more quickly and directly. |
| 28 | My understanding was that it was used to clean the toxins from the blood system. This is a slower machine than all the hospitals have. His blood pressure couldn't take the stress of the other machine. |
| 29 | This machine is being used to remove water from my body because I was told that my blood volume was too high so it needed to be removed. |
| 30 | To clean toxins out of my father's body |
| 31 | To keep his kidneys working properly |
| 32 | It has something to do with removing water and blood from the kidneys |
